# Investigating the Acceptability of an Interactive Television Intervention Promoting Social Links Among Older Adults Living at Home and in Care Institutions: Qualitative Interview and Questionnaire Study

**DOI:** 10.2196/58051

**Published:** 2024-12-02

**Authors:** Bérangère Naudé, Anne-Sophie Rigaud, Maribel Pino

**Affiliations:** 1 Faculté de Médecine Université Paris Cité Maladie d’Alzheimer Paris France; 2 Broca Living Lab CEN STIMCO Paris France; 3 Service Gériatrie 1&2 Centre Mémoire de Ressources et Recherches Ile de France AP-HP, Hôpital Broca Paris France

**Keywords:** interactive television, iTV, acceptance, older adults, nursing home, residential facility, technology acceptance model, TAM, mobile phone

## Abstract

**Background:**

When older adults (OAs) can no longer live independently at home, they have the option to choose from various types of geriatric care institutions, such as residential facilities or nursing homes. For several years now, thanks to the development of interactive television (iTV), social link functions have been accessible directly on televisions, tools that are already integrated into residents’ rooms. The acceptance of technologies specifically targeting older users, as well as iTV, has been widely documented in the literature, incorporating factors from the innovation resistance model.

**Objective:**

This research aims to enrich the acceptance of existing models of innovation by OAs living in different settings.

**Methods:**

User tests were carried out to evaluate OAs’ experiences with iTV and identify the factors involved in its acceptance. A total of 32 OAs living at home, in nursing homes, or in residential facilities in France were interviewed between November 2022 and June 2023. iTV acceptance was examined using an interview grid based on the technology acceptance model and included the following factors: intention to use, perceived usefulness, perceived ease of use, user resistance, anxiety, facilitating conditions, and user characteristics.

**Results:**

The deductive qualitative analysis based on the technology acceptance model helped to identify 33 concepts.

**Conclusions:**

This study has contributed to the literature on the acceptance of iTV by OAs living at home and in geriatric institutions, particularly by enriching existing models and proposing new avenues for reflection.

## Introduction

### Background

Older adults (OAs) experience perceptual, physiological, and cognitive changes with aging [1,2], which may affect their daily independence. When it is no longer possible for them to remain at home (due to, eg, the presence of cognitive problems, functional problems, or a combination of both cognitive and functional problems) [3], OAs may choose between different types of accommodation, including residential facilities (RFs) or nursing homes (NHs). In France, RFs represent a step between home and institutional care [4]. For several years now, and especially since the COVID-19 pandemic, NHs and RFs have been equipped with information and communication technologies (ICTs; eg, tablets, computers, and smartphones) to promote a feeling of connection between residents and their relatives [5,6]. However, access is not the same as accessibility. In fact, OAs appear to experience stress when using technology, also known as technological anxiety, which is described as (free translation) “a negative psychological state associated with the use or fear of having to use ICT. This experience produces feelings of anxiety, mental fatigue, skepticism and inefficiency” [7]. OAs who are anxious and lack confidence in their own abilities also show greater resistance to gerontechnologies [8-10] and an increased need for support.

With the development of interactive television (iTV), social networking functions (eg, social networks, video calls, and text messages) are now accessible directly on the television screen [11,12]. The appeal of this new technology, in contrast to other ICTs, is rooted in its familiarity. Given that television systems are already a fundamental component of OAs’ daily routines (ie, their main source of information and entertainment) [12], iTV may offer a less anxiety-inducing alternative to traditional ICTs [12-16]. Although iTV seems to be able to facilitate access to social link functionalities within geriatric institutions, its long-term use depends on several other factors, including organizational (eg, staff turnover and additional workload), human (eg, health, self-efficacy, and technological and social habits), ethical (eg, privacy and agism), and technological factors (eg, technical and ergonomic problems with iTV and nonexistent or inadequate training) [17-19]. The literature on the acceptance of technologies by OAs is often stereotypical [20], considering the older user to be resistant to any form of innovation and lacking motivation to use a technology [21]. However, some authors [21] challenged these studies, explaining that while OAs value their independence, privacy, and social interaction, products designed specifically for this population focus mainly on safety and assistance aspects. In the technology paradoxes framework [22], consumers face paradoxes while using technology, creating negative emotions, such as anxiety and stress. To combat these emotions, consumers tend to adopt various pre- or postacquisition coping strategies to avoid or confront the technology. Applying this framework to older consumers, Wilson-Nash and Tinson [23] found that most of the strategies adopted by OAs are confrontational, showing that despite previous assumptions of lack of dynamism or willingness [24,25], OAs are keen to master the technology. There is a wealth of literature on the factors influencing the adoption of technological innovations, and several explanatory models have been developed. The 2 main ones are the technology acceptance model (TAM) proposed by Davis [26] and the unified theory of acceptability and use of technology (UTAUT) proposed by Venkatesh et al [27].

### Models of Technology Acceptance

According to the TAM, the acceptance of technology, that is, the extent to which a product will be used or not, depends on 2 main factors: perceived usefulness (PU) and perceived ease of use (PEOU) [26]. Subsequently, other extended versions of the TAM emerged, culminating in the proposal of an integrative model named UTAUT [27]. Venkatesh et al [27] differentiate between the determining factors, that is, factors influencing the intention to use (IU) or the use of technologies, and the moderating factors, that is, factors influencing the determining factors. These include expected performance; expected effort; social influence; and the presence of facilitating conditions (FCs), such as documentation. Although these models have been used in a variety of contexts, their original field of application is the workplace. Venkatesh et al [28] then proposed a second version of the UTAUT (UTAUT 2), which is better adapted to the context of consumer use. Three other factors have been added to the existing determining factors: hedonic motivation, monetary value, and habit. Finally, these same authors proposed ways of developing the model, such as integrating contextual factors (eg, organization and physical environment) and individual factors (eg, user and technology characteristics) [29]. At the same time, Chen and Chan [8] have been looking at the acceptance of technologies by OAs and have developed the Senior TAM. This model incorporates factors from both TAM and UTAUT, such as PU, PEOU, attitude to use, FCs, self-efficacy, and anxiety about gerontechnologies. The authors also added age-related factors such as perceived health, cognitive ability, attitude to aging, satisfaction with life, social relationships, and level of physical functioning. Age, gender, level of education, and economic status were also considered as control variables.

### Innovation Resistance

The adoption of a technological innovation may also depend on the user’s degree of resistance to change, that is, to the maintenance of the status against the pressure of change [30]. Any disruption to users’ routines can alter their psychological equilibrium, which then needs to be adapted to reduce resistance [31,32]. An innovation can only be adopted once the initial resistance has been overcome by users [33]. Several authors have attempted to identify the factors influencing this resistance, such as the practice habit, and the perceived risk of the innovation [34] or the innovation, propagation mechanism, and user characteristics (UCs) [33]. According to Joseph [35], 3 types of factors seem to influence resistance: functional, psychological, and informational factors. Finally, user resistance (UR) seems to depend on several multidimensional factors.

### The Acceptance Model Used in This Study

In their study on the acceptance of iTV, Im et al [36] proposed an integrative model combining the models of acceptance (TAM [26]) and resistance to innovation [33]. They aimed to go beyond previous theoretical frameworks to better explain the dynamic nature of adoption [37]. According to these authors, the factors influencing iTV adoption are PEOU, PU, UCs (eg, previous experience with innovations and self-efficacy), resistance to innovation, and IU. However, this model does not consider the characteristics of OAs (eg, cognitive and physical disorders, lack of digital literacy, technological anxiety, and low sense of self-efficacy) [2,38,39], nor does it consider the specific characteristics of geriatric residents [40].

This paper presents a qualitative study investigating the factors that might influence the acceptance of the iTV system by OAs living at home, in RFs, and in NHs. The first step was then to create an acceptance interview grid addressing both the specific features of the technology and those of the population studied. Therefore, this study aimed to enrich existing models of acceptance by suggesting new lines of thought on the determinants of acceptance proposed in these models.

## Methods

### Overview

The method used was borrowed from the user-centered design and the qualitative and quantitative methods of user testing [41]. The aim was to place participants in real-life conditions based on pre-established iTV use scenarios. By combining observation and interviews with the participants, the researcher was able to identify the difficulties encountered when using the technology. As part of this study, the participants could receive cues if they were stuck, asked for help, or made a mistake.

### Materials

#### The iTV

**Figure 1:**
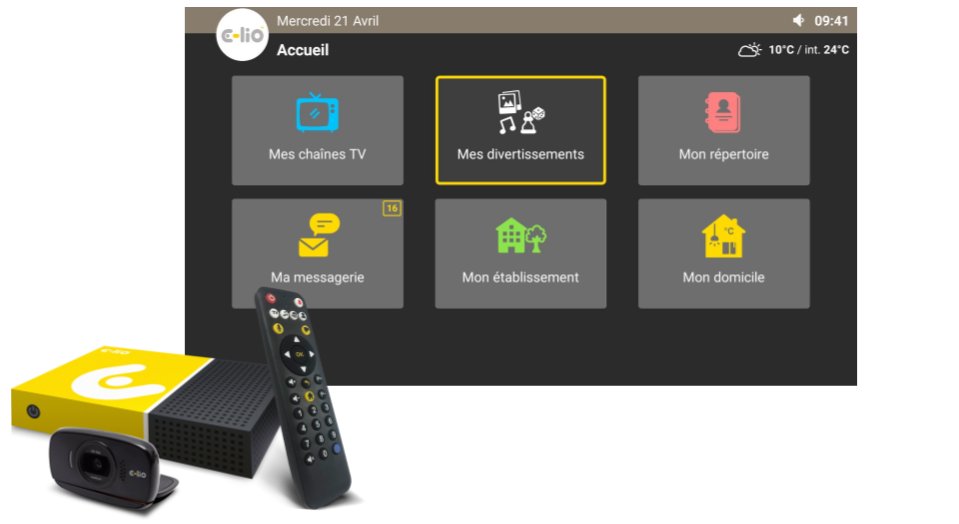
The e-lioTV system and the main menu interface: the box, camera, and remote control.

The iTV selected for the user tests (e-lioTV) is a system developed by Technosens [42] and installed in geriatric institutions since 2011. A total of 53 NHs and RFs in France are equipped with this iTV, which is installed directly in residents’ bedrooms. This iTV consists of a box connected to a television screen and offers communication (eg, text messages, photos, and video calls); entertainment (eg, television channels and radio); and information services (eg, news from the institution; Figure 1). All these functions can be accessed via a single remote control.

#### Questionnaires and Interview Grid

To identify the different profiles of participants, we administered a sociodemographic and a technological habit (eg, frequency of use of technologies) questionnaire. A semistructured interview to examine iTV acceptance was conducted at the end of the user tests. An interview guide (Table 1) was constructed based on the literature on the acceptance of technologies [26,43,44], and in particular iTVs [36], among OAs [8]. The final extended TAM (e-TAM) consists of the following seven dimensions: (1) IU; (2) PU; (3) PEOU; (4) UR; (5) anxiety; (6) FCs (eg, peer support and self-efficacy); and (7) UCs (eg, previous experience and interest in technology, social influence, and perceived health). Participants were asked to give their opinion on statements using a 5-point Likert scale (“1” indicating “strongly disagree,” “2” indicating “disagree,” “3” indicating “neutral,” “4” indicating “agree,” and “5” indicating “strongly agree”). We then asked them to explain their responses.

**Table 1:** The interview guide based on the extended technology acceptance model.

Dimensions and affirmations			References
Perceived utility	“E-lioTV is practical for me.” “E-lioTV provides me with various useful functions and services.”	[36]	
Perceived ease of use	“It takes much time and effort to understand and use e-lioTV.” “I found e-lioTV easy to use.”	[26,36]	
User resistance	“Using e-lioTV is burdensome.” “E-lioTV is not for me.”	[36]	
Anxiety	“I’m afraid of using e-lioTV.” “I’m afraid that if I press the wrong button on the remote, I might break something on e-lioTV.”	[43]	
Intention to use	“I want to have e-lioTV in my home.” “I would recommend others using e-lioTV.”	[36]	
Facilitating conditions	“In general, if I have a problem with a device, I ask my family or care staff for help.” “It is easy for me to understand and use new devices.”	[36,44]	
User characteristics	“The people I care about push me to use messages and video calls.” “In general, I’m curious to learn how to use a new device.”	[44,45]	

### Participants and Recruitment

Participants of the study lived in geriatric institutions (eg, NH and RF) or at home and were not e-lioTV users. Participants living at home were recruited in Paris, while others were recruited in the Saint-Etienne and Grenoble regions. The inclusion criteria were that the participants should be (1) aged >60 years, (2) literate, and (3) able to use a television remote control by themselves. Exclusion criteria were (1) having a Mini-Mental State Examination score of <16 [46] and (2) having visual, hearing, or motor problems that prevented them from using a television remote control, unless they could be compensated for by a technical aid. To this end, for NH and RF residents, professionals were asked to draw up a preliminary list of willing residents who met the above criteria.

In total, 38 participants were approached (RF: n=10, 26%; NH: n=12, 32%; and home: n=16, 42%) between November 2022 and June 2023. Before each scenario, the researcher asked whether the participant wished to continue. Of 38 participants, 5 (13%) chose to stop before the end of the test due to increasing fatigue and 1 (3%) person was excluded because he was illiterate. A total of 32 participants were included, including 23 (72%) women and 9 men (28%). The demographic characteristics of the 3 groups are presented in Table 2. These data seemed in line with the literature, with participants living in NH belonging almost entirely to the category of those aged >80 years [4]. In this study, it is interesting to note that almost all the participants living at home had a higher education degree (postgraduate degree) compared with the institutionalized participants.

**Table 2:** Sociodemographic characteristics of participants (N=32).

	Gender, n (%)		Age (y), n (%)				Education, n (%)	
	Women (n=23)	Men (n=9)	60 to 69 (n=5)	70 to 79 (n=11)	>80 (n=16)	Undergraduate (n=17)		Postgraduate (n=15)
NH^a^ (n=7)	4 (17)	3 (33)	0 (0)	1 (9)	6 (38)	7 (41)		0 (0)
RF^b^ (n=9)	7 (30)	2 (22)	3 (60)	1 (9)	5 (31)	8 (47)		1 (7)
Home (n=16)	12 (52)	4 (44)	2 (40)	9 (82)	5 (31)	2 (12)		14 (93)

^a^NH: nursing home.

^b^RF: residential facility.

To explore participants’ technological expertise, we assessed the frequency of mobile phone use by functionality (eg, calling, answering a call, sending a text message, and checking a text message) and the overall frequency of computer and tablet use. We then categorized their expertise with the technology as follows: a person is considered an “expert” if they use the feature or technology “several times a day” or “every day or almost every day,” and a person is considered a “nonexpert” if they do not use the feature or technology or if they use it “every week” or “every month.” According to Table 3, most participants (RF: 9/9, 100%; NH: 6/7, 86%) in the RF group and NH group were not experts in the use of computers, unlike participants in the home group. In addition, some of the participants in the RF group (make a call: 5/9, 55%; send a text message: 4/9, 44%) used their mobile phones more frequently than those in the NH group (make a call: 1/7, 14%; send a text message: 0/7, 0%). It is interesting to note that among the smartphone users (NH: n=0, 0%; RF: n=6, 67%; and home: n=15, 94%), those living in RF tended to restrict the use of their mobile phones to calling and messaging.

**Table 3:** Technological expertise of participants (N=32).

Level of expertise			Mobile phone, n (%)									Computer, n (%)			Tablet, n (%)
			Make a call		Answer a call		Send a text message		Receive a text message						
**NH^a^ (n=7), n (%)**															
	Expert	1 (14)		2 (29)		0 (0)		0 (0)		1 (14)			0 (0)		
	Nonexpert	6 (86)		5 (71)		7 (100)		7 (100)		6 (86)			7 (100)		
**RF^b^ (n=9), n (%)**															
	Expert	5 (56)		8 (89)		4 (44)		5 (56)		0 (0)			1 (11)		
	Nonexpert	4 (44)		1 (11)		5 (56)		4 (44)		9 (100)			8 (89)		
**Home (n=16), n (%)**															
	Expert	14 (88)		15 (94)		15 (94)		15 (94)		16 (100)			5 (31)		
	Nonexpert	2 (12)		1 (6)		1 (6)		1 (6)		0 (0)			11 (69)		
**Total (N=32), n (%)**															
	Expert	20 (62)		25 (78)		19 (59)		20 (62)		17 (53)			6 (19)		
	Nonexpert	12 (38)		7 (22)		13 (41)		12 (38)		15 (47)			26 (81)		

^a^NH: nursing home.

^b^RF: residential facility.

### Procedure

The tests were carried out individually and systematically by 2 people (BN and a colleague). While the first author conducted each part of the procedure, the other researcher was invited to observe and take notes. Informed consent was obtained on the day of the appointment after the participant had been reminded of the objectives of the research. Before starting the scenarios on the iTV, the overall cognitive level of each participant was assessed, either by the institution’s psychologist or by the researcher (first author), using the Mini-Mental State Examination. Then, participants were asked to complete a sociodemographic questionnaire and to share their technology use habits. After a brief presentation of the principle and overall functioning of the iTV (eg, the different components of the tool, such as the box, camera, and remote control, and the main features), participants’ first impressions were gathered. The iTV was used through 3 scenarios of increasing difficulty, each with more steps than the last (eg, answering a video call, checking a text message, and making a video call). The researcher then collected participants’ opinions or recommendations on the iTV throughout the scenarios. Once the 3 scenarios had been completed, the semistructured interview, based on the e-TAM, was conducted. If necessary, the researcher asked participants to develop some answers. Finally, and at the participant’s request, the researcher could also provide an additional explanation of the iTV’s marketing status and target audience, as well as the rest of its functionalities not used in this test (eg, radio, news from the institution, and games). Participants were then invited to react on their behalf, as well as on behalf of someone close to them.

### Ethical Considerations

Ethics approval for this study was obtained from the Université Paris Cité Research and Ethics Committee in November 2021 (approval number 00012021-91). Validation of the data management procedures and related compliance with the General Data Protection Regulation was obtained from the data protection office and registered in the general register of Greater Paris University Hospitals (Assistance Publique-Hôpitaux de Paris) in February 2022 (20220228123925). The collected data were anonymized in such a way that identification of individuals or sources of information is not possible. Participation in the study was voluntary and subject to the signing of an informed consent form. Participants were informed of their right to withdraw from the research project (resulting in the deletion of their data) and their right to access and rectify information concerning them.

### Qualitative Analysis

Two authors participated in the qualitative analysis (BN and ASR). Deductive thematic analysis was carried out based on the transcribed user tests. The aim was to identify the factors that could influence performance on the iTV and, therefore, its acceptance, based on the e-TAM factors. To this end, we took inspiration from the Qualitative Analysis Guide of Leuven [47] following three preliminary steps before coding the interviews: (1) highlighting important elements and writing a mini report on the participant’s characteristics, (2) designing conceptual schemes, and (3) validity testing. Once the conceptual schemes had been tested and validated, we were able to draw up a list of concepts used to code the transcripts. These concepts were classified according to the e-TAM factors, allowing us to develop some of them further or even to create new ones. An overall conceptual framework for our transcripts, in the form of a mind map, was thus created. Finally, each concept was described, including a short summary of the points raised in the interviews and a few key verbatims.

## Results

### Overview

**Figure 2:**
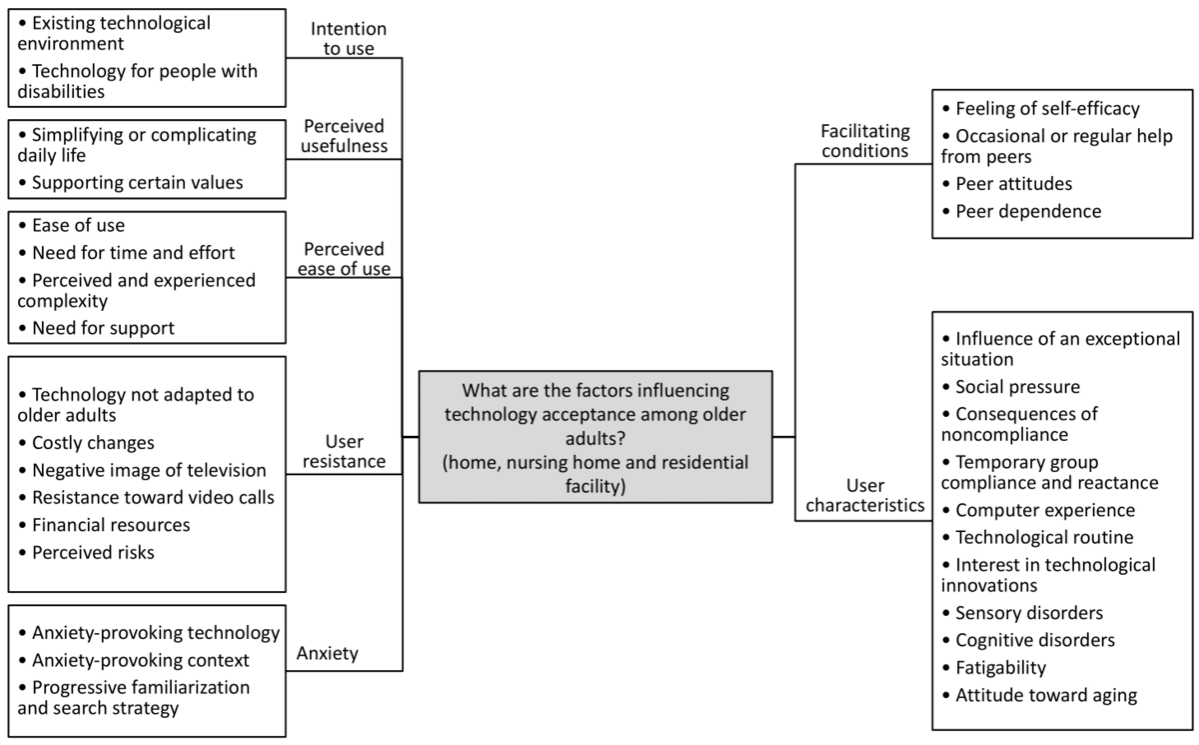
Mind map of themes and subthemes from the deductive qualitative analysis based on the extended technology acceptance model.

**Figure 3:**
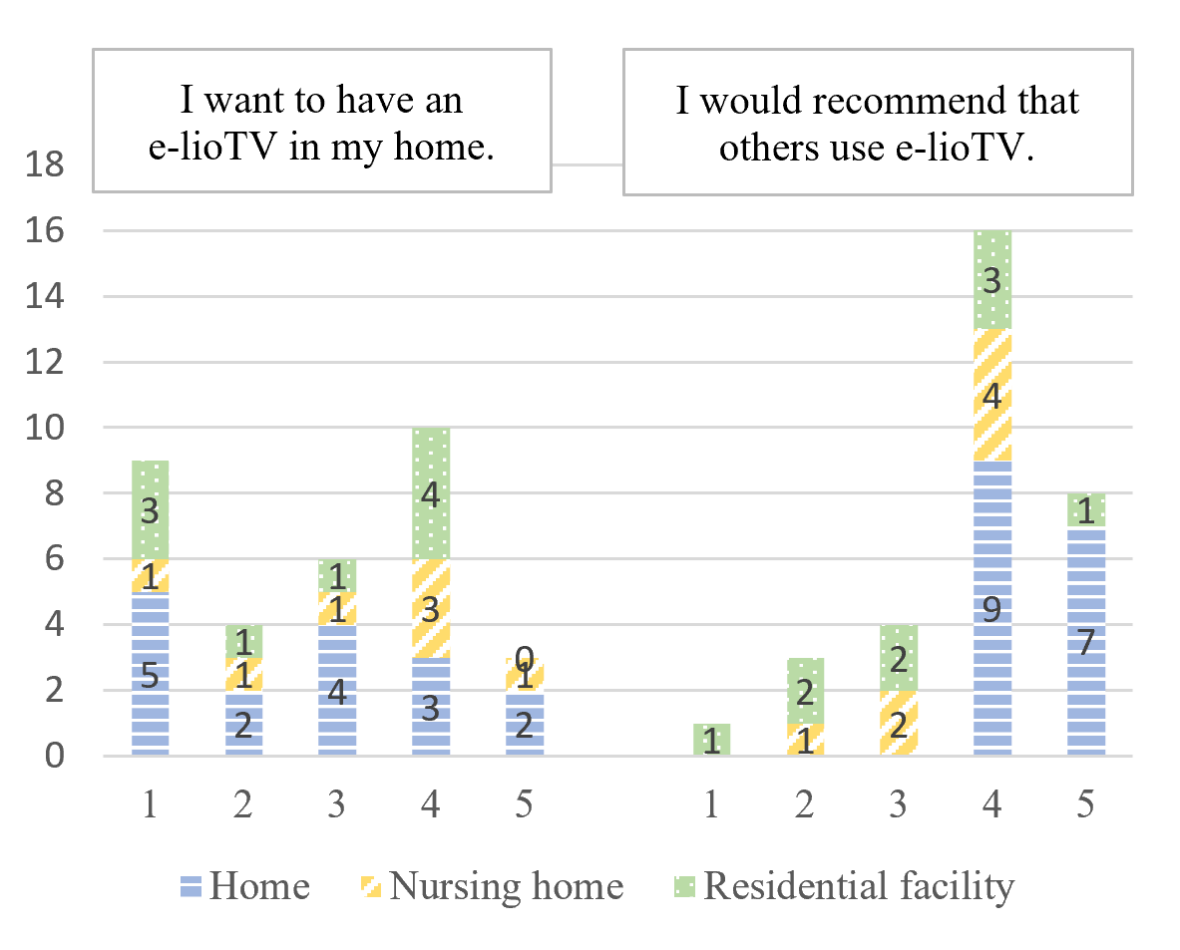
Intended use of interactive television by older adults living at home, in residential facilities, and in nursing homes (1: strongly disagree; 5: strongly agree).

**Figure 4:**
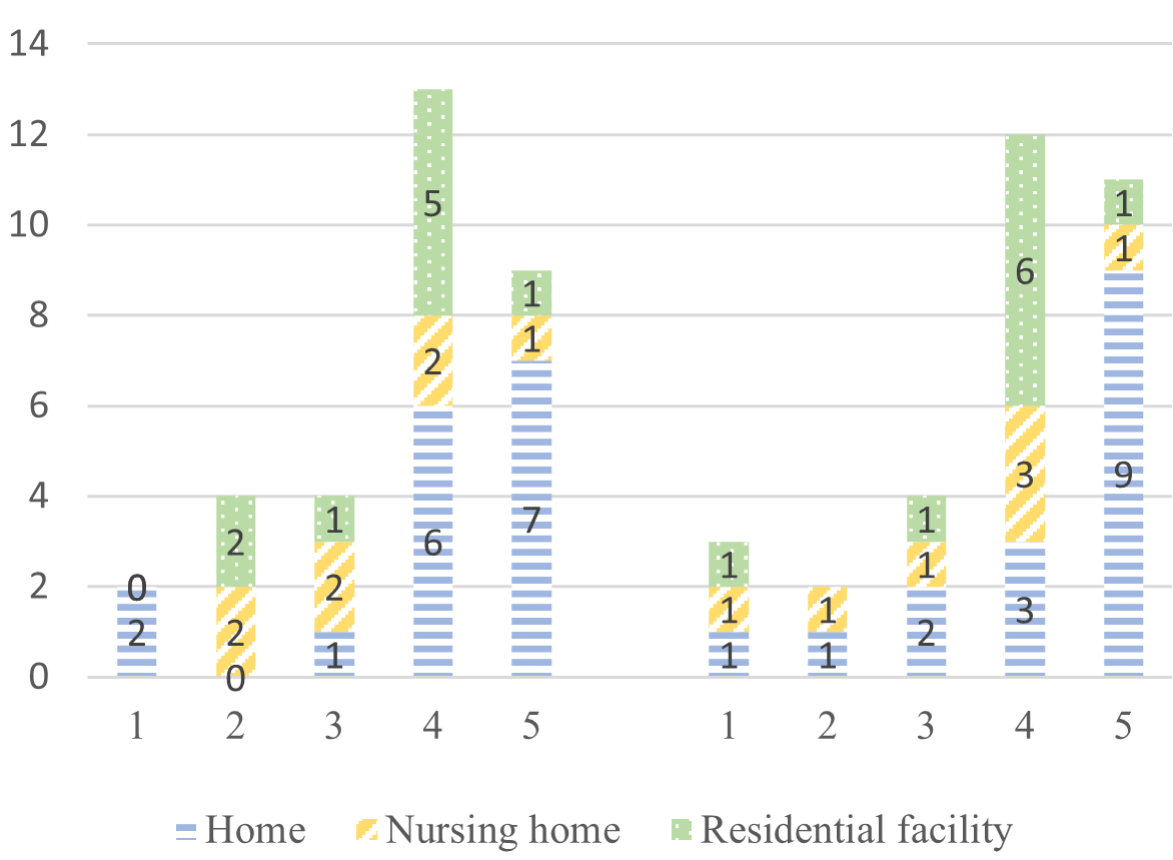
Perceived usefulness of interactive television by older adults living at home, in residential facilities, and in nursing homes (1: strongly disagree; 5: strongly agree).

**Figure 5:**
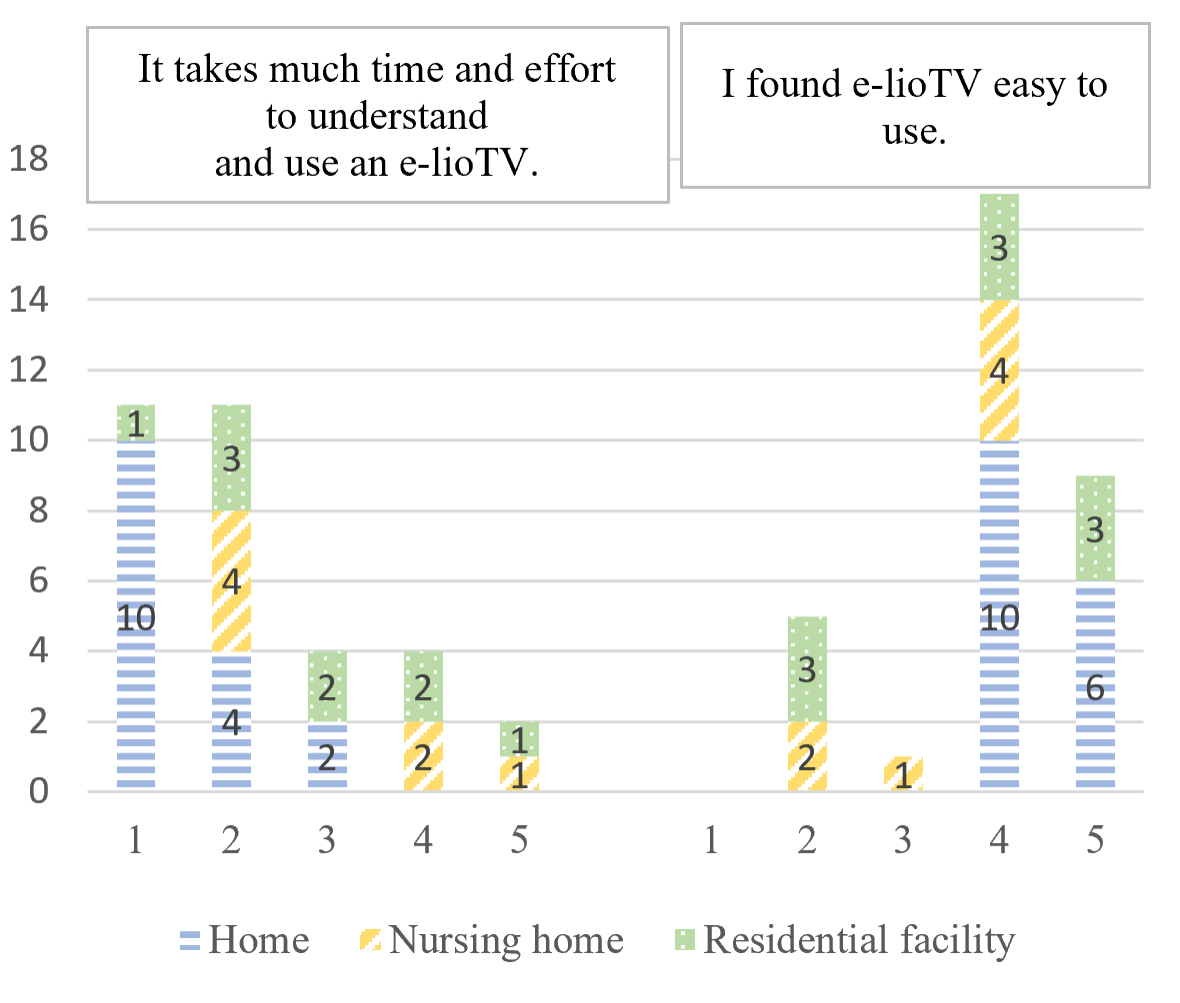
Perceived ease of use of interactive television by older adults living at home, in residential facilities, and in nursing homes (1: strongly disagree; 5: strongly agree).

**Figure 6:**
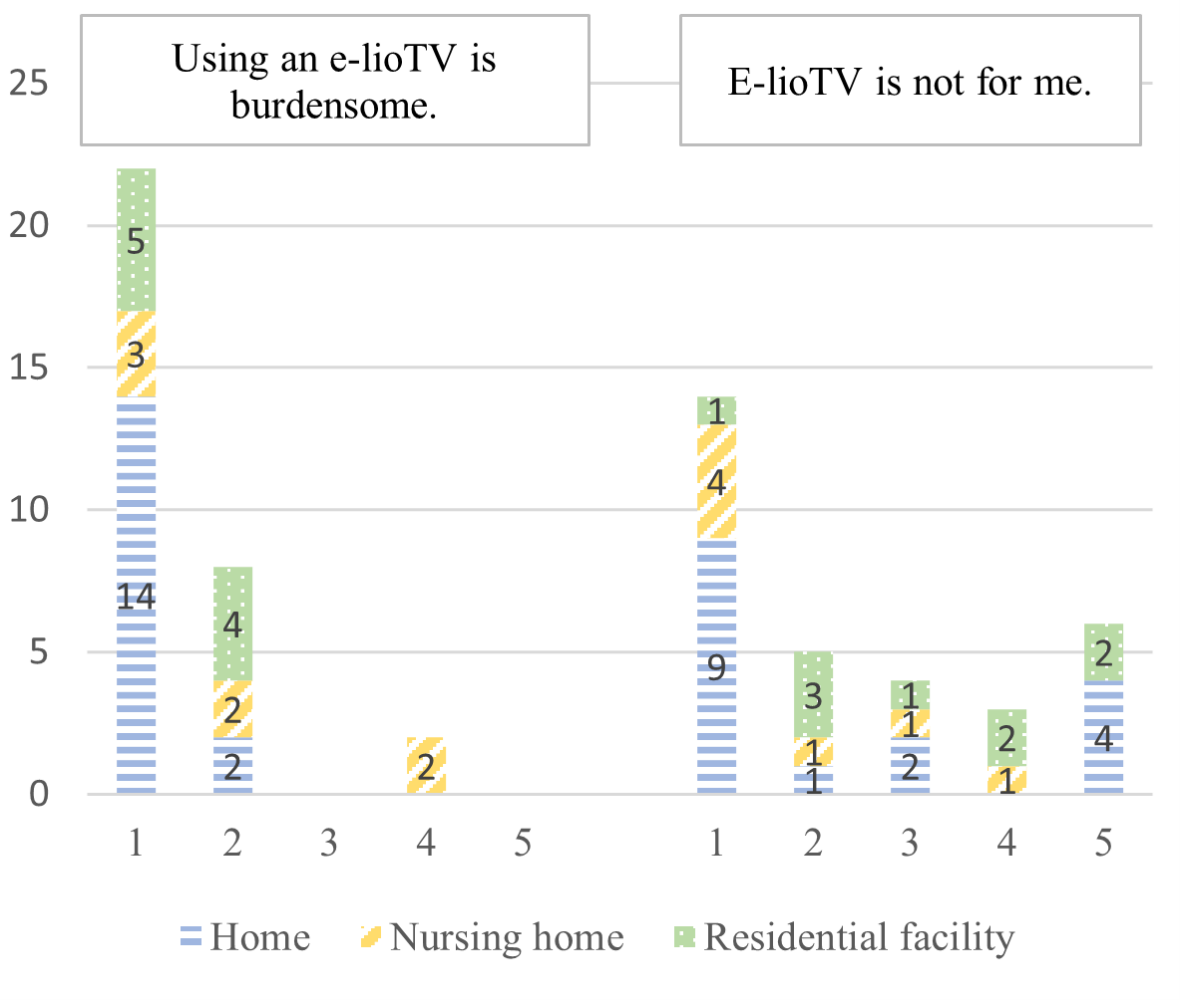
User resistance toward interactive television by older adults living at home, in residential facilities, and in nursing homes (1: strongly disagree; 5: strongly agree).

**Figure 7:**
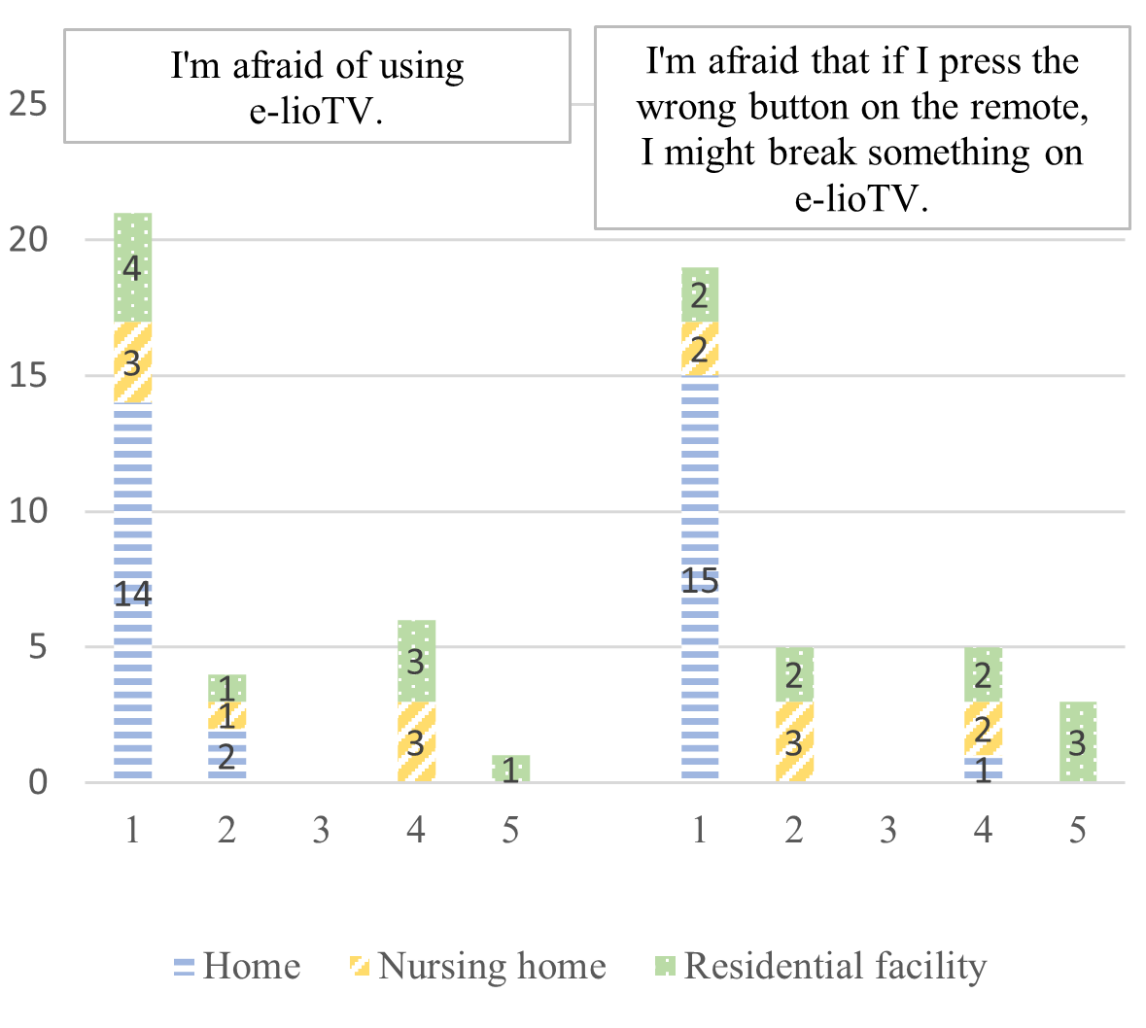
Anxiety while using interactive television by older adults living at home, in residential facilities, and in nursing homes (1: strongly disagree; 5: strongly agree).

**Figure 8:**
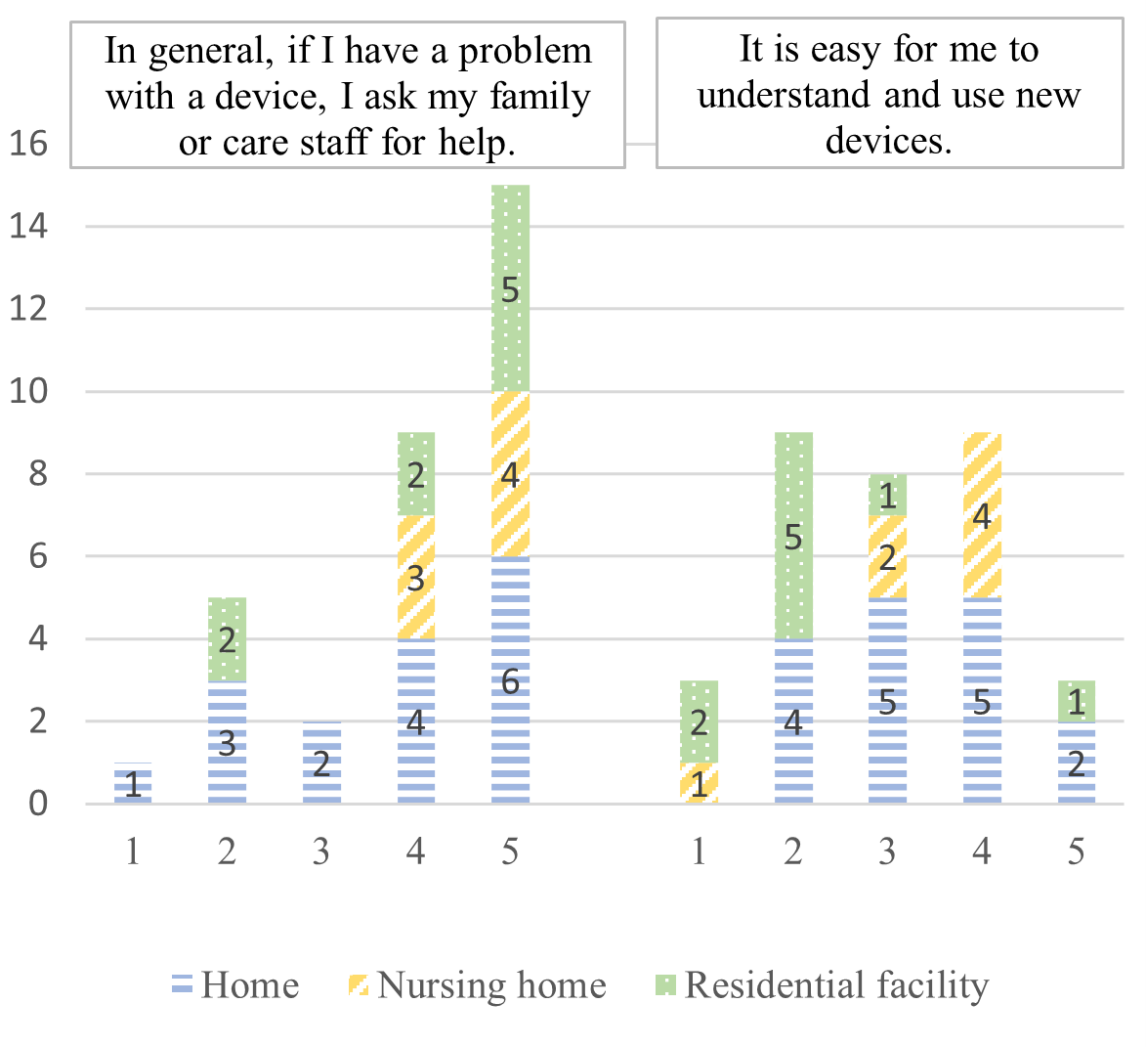
Facilitating conditions for the use of interactive television by older adults living at home, in residential facilities, and in nursing homes (1: strongly disagree; 5: strongly agree).

**Figure 9:**
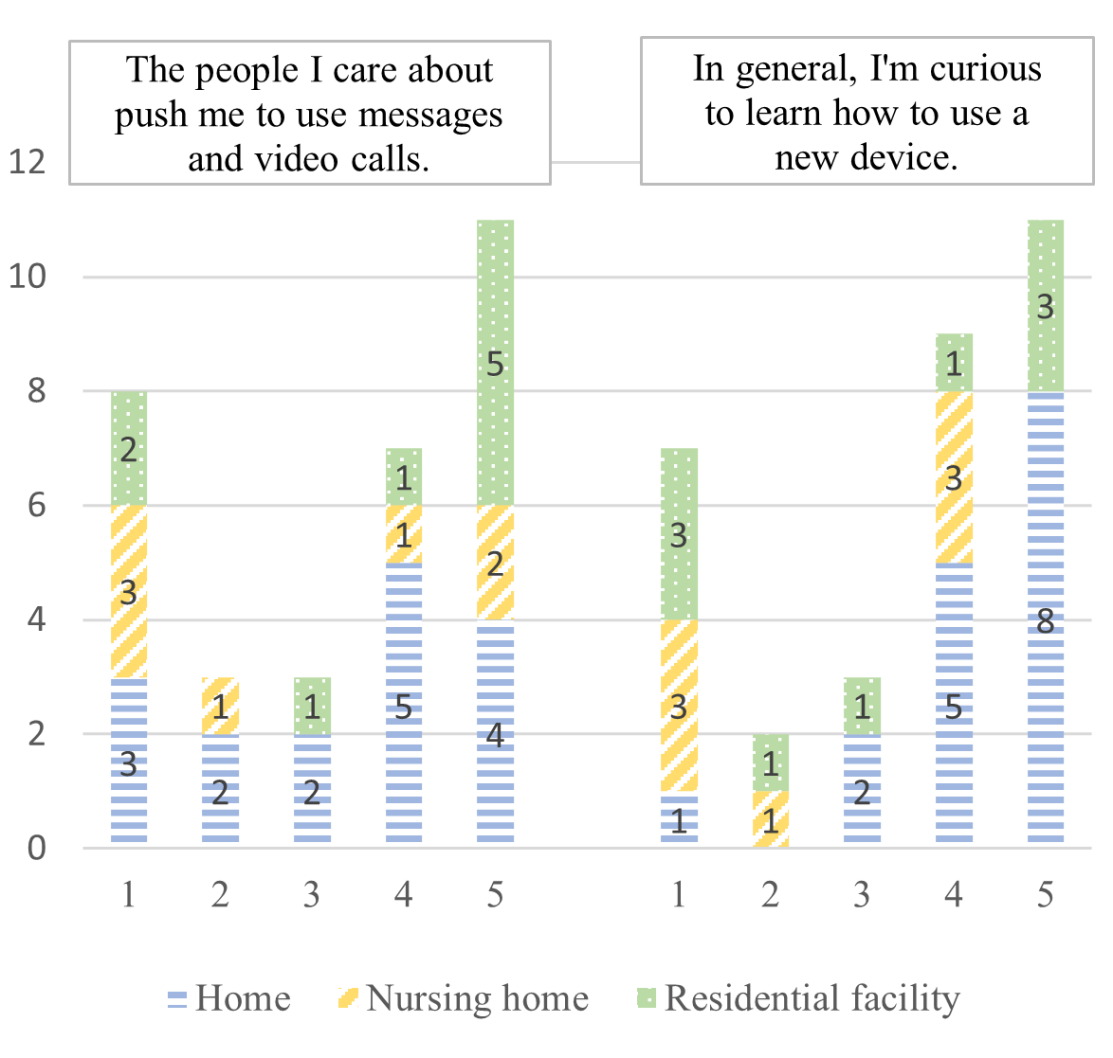
Participants’ characteristics (1: strongly disagree; 5: strongly agree).

According to the e-TAM, iTV acceptance depended on 7 factors: IU, PU, PEOU, UR to iTV, anxiety when using iTV, FCs, and UCs. A summary of the 33 concepts found during the deductive qualitative analysis is presented in Figure 2. Figures 3-9 show the number of participants who rated 1, 2, 3, 4, or 5 on the Likert scale. Different colors and symbols are used to represent the 3 living environments.

### IU Dimension

#### Overview

Although they seemed to perceive an interest in iTV in general, participants were equally divided between those who would like to have an iTV at home (13/32, 41%) and those who would not (13/32, 41%). However, all participants living at home would still recommend iTV to their friends and family (Figure 3).

#### Existing Technological Environment

Many participants (30/32, 94%) already owned several technologies, some for several years, and had well-established communication and information habits. Some of them (6/32, 19%) saw no point in using an iTV as a complement. The technology was perceived as a gadget with no added value compared to existing technology (fewer functions or accessibility options, such as voice):

Me personally? No, no, I won’t use it....Because I have other things, things that react to my voice, things that are much more responsive.

Participant 2, home group

There was a multiplicity of everyday technologies:

I have enough machines to do it.

Participant 13, home group

It is interesting to note that this barrier only concerned OAs living at home, that is, in this study, the participants who owned the most technology.

#### Technology for People With Disabilities

E-lioTV could be seen as a technology for people who are losing their independence with temporary or permanent cognitive or mobility impairments. In fact, one of the advantages of the iTV identified was the centralization of functions on a technology that is already used on a daily basis and located in a single room, often the most frequented in the home. In the event of a call, this could reduce the risk of falling (getting up in a hurry if the phone rings):

It’s certain that if one day I’m a lot less mobile, and if I’m less able to flit from one thing to another, I’ll use this device.

Participant 2, home group

However, this comment was only made by OAs living at home (7/32, 22%), and if we observe the left side of Figure 3, no link can be made between living settings and the IU.

### PU Dimension

#### Overview

The iTV seemed practical for most participants living at home (13/16, 81%) and in RF (6/9, 67%), with the services offered perceived as useful (Figure 4). However, the opinions of the participants living in NHs seemed to be more nuanced.

#### Simplifying or Complicating Daily Life

Most participants (22/32, 69%) had a very positive attitude toward iTV and were satisfied with the principle of having a television screen that brings together several functions (23/32, 72%). According to 1 participant, this principle is “very clever” (Participant 3, home group) and avoids the multiplication of technologies in the home (10/32, 31%). Using a television system also means that functionalities such as games can be accessed on a larger screen. Furthermore, it is easier to position oneself in front of a camera on a television screen than on a smartphone. However, some participants (4/32, 12%) seemed to consider technological innovations as an unnecessary complication of everyday life. For example, even before the operation of the iTV was explained to them, some participants showed no interest in discovering this technology:

I don’t need it because I just want to keep watching the news and that’s all...I’m starting to say: “Whoa, let’s just stay as we are, let’s not complicate our lives.”

Participant 6, NH group

The value of technology can also be linked to a person’s lifestyle and job. One former farmer, who had always lived in a rural environment, had never understood the usefulness of a computer:

And then, in the countryside, it’s not really that useful. The main thing is to have the essentials.

Participant 14, NH group

#### Supporting Certain Values

According to some participants (12/32, 37%), the iTV’s features were relevant because they supported values that were important to them, including connection with the outside world; the iTV could facilitate interaction with loved ones and provide people with the opportunity to take part in moments of life through photos and video calls:

Oh yes, indeed, you can travel that way.

Participant 7, NH group

Autonomy and health was also important; other participants appreciated the reflection involved when using the iTV, as well as the games:

That way, he can do cognitive remediation on his own.

Participant 3, home group

Video calls could also help to reassure family and friends.

### PEOU Dimension

#### Overview

Most participants found the iTV easy to use (NH: 4/7, 57%; RF: 6/9, 67%; and home: 16/16, 100%). However, a few participants living in RFs (3/9, 33%) and NHs (3/7, 43%) reported that they needed a little more time to learn how to use it (Figure 5).

#### Ease of Use

The iTV seemed easy to use for most participants (26/32, 81%), even for those with no computer skills or with mild cognitive impairment. Several participants (14/32, 44%) found that the remote control had few new buttons, making it easy to identify the most important ones. The icons also seemed logical and easy to understand. The information inserted on the television screen served as a memory aid; instead of spending time looking for the solution, they felt that the system was doing the work for them by pointing out the right buttons, something that is all the more relevant for OAs:

Well yes, everything has been spoon-fed, you know.

Participant 1, home group

It’s visual, but maybe you need that more when you’re older too.

Participant 14, home group

This guidance greatly reduced the amount of information to be memorized, facilitating familiarization with the system and success during the first moments of interaction, an important phase in forming a good first impression. Finally, the participants had the feeling that they were being supported:

It’s pretty simple, isn’t it, because you’re really being taken by the hand from the beginning to the end.

Participant 1, home group

Finally, 1 participant was used to a certain complexity on a daily basis (2 remote controls) and seemed pleasantly surprised to be able to do so many things with just 1 remote control.

#### Need for Time and Effort

Although the iTV was considered rather easy to use, some participants (17/32, 53%) said that they needed time to get used to it. This time was needed to develop automatisms and reflexes and to become familiar with the buttons and the iTV’s operating principle, that is, looking at the screen to find the right button:

It requires some adaptation, and adaptation takes a little longer now.

Participant 11, home group

However, although learning and problem-solving take longer with age, participants were not bothered, also because they had more time to spend on this than young adults:

It took me a little while to find it, but does it need to be immediate? I’m not sure.

Participant 8, home group

Regarding the need for effort, several participants (6/32, 19%) emphasized the cognitive resources required to handle the iTV. Using this tool seemed to have a cost in terms of attention and mental flexibility to make the link between the guidance on the screen and the buttons on the remote control, select the relevant information on the screen, and remember the actions of certain buttons:

I was doing gymnastics between looking at the screen, looking at the remote control.

Participant 6, NH group

#### Perceived and Experienced Complexity

Some participants (9/32, 28%) experienced difficulties in learning how to use the iTV, such as recognizing the icons on the remote control, pressing the buttons gently, or identifying the actions to be performed on the television screen. One participant stressed the need to simplify the technology as much as possible to avoid discouraging users:

It can be very discouraging for someone who doesn’t understand it very well, and maybe not curious enough to insist either.

Participant 14, home group

Indeed, when faced with several failures, 1 participant was tempted to give up:

Pfff. What am I going to do? I can’t do anything. I’m stuck.

Participant 15, RF group

Beyond the experienced complexity, the perceived complexity could influence the way the iTV was approached. Indeed, even before using the device for the first time, participants had already formed an initial impression based on their personal experience with their television. Some participants (4/32, 12%) perceived the remote control as complex (those who were using a remote control with fewer buttons on a daily basis), while others imagined that the iTV would be simple to use, similar to an ordinary television. But this first impression could be misleading, particularly because of the difficulty of applying knowledge of other technologies to this device:

It looks very simple, but in the end, you have to find out what it means on the remote control.

Participant 14, home group

I don’t know why...I end up thinking about how to use a remote control.

Participant 14, home group

#### Need for Support

Most participants (14/32, 44%) said that they needed extra support or even guidance to be able to use the iTV. The fact that there were no instructions and that they had to manage on their own from the start seemed to disconcert some of them:

We’re letting people get started without giving them a user manual?

Participant 2, home group

One participant highlighted the necessity for OAs to record all details in writing. A summary note of the remote control with the actions associated with each button might be enough, particularly in the early stages of the interaction. However, 1 participant was reluctant to have a user manual, considering it too complicated in general. Another participant preferred to have support from someone, particularly to reassure her during the first moments of interaction:

Well, at least.... I don’t know, one or two days to fully understand the buttons.

Participant 15, RF group

### UR Toward the iTV

#### Overview

Although participants did not seem to find the iTV use burdensome (30/32, 94%), almost half of them (13/32, 41%) felt that iTV was not adapted to their needs and uses or did not give an opinion (Figure 6).

#### Technology Not Adapted to OAs

Some participants (7/32, 22%) immediately thought that the iTV was not suited to them or their NH neighbors for various reasons, including health conditions, with a participant stating, “We’re all a bit lost, I don’t think it’s really suited to our home” (participant 6, NH group). Other reasons included poor knowledge of technology use and age. This last point can be seen as self-agism. One participant seemed outraged by this stereotype:

[This television is not for me] Why? Because I’m stupid? And I’m old? [Laughs].

Participant 7, NH group

Finally, although participants found the functionalities rather useful, they almost systematically considered that the iTV was made for someone else, seeing no point in changing their habits.

#### Costly Changes

Some participants (4/32, 12%) had a negative attitude toward application updates or the switch to smart televisions, showing annoyance or even stress when using them. OAs found it difficult to adapt to change, with each new feature requiring an effort to understand and learn, which became even more demanding with age (eg, slowness and need for repetition):

That’s what’s annoying, it’s the constant updates that turn everything upside down. So, you make an effort to keep up with it, you start to assimilate it more or less and that’s it, off you go again.

Participant 12, home group

So, although the television remote control is used on a daily basis, most participants (5/32, 16%) were not used to using other buttons apart from the television channels to keep control of the situation. Therefore, using an iTV necessarily requires a learning curve. Another explanation for this annoyance could be the imbalance between the cost and benefit of the change, as it was not necessarily perceived as an improvement, especially if it made other functions less accessible. One participant showed particular resistance, mainly because using iTV made it harder for her to access television channels.

#### Negative Image of Television

A few participants (5/32, 16%) said that they disliked television in general, preferring to use another technology (eg, computer) or do another activity. The television system is generally perceived as a technology that isolates people and encourages immobility. In addition, 2 participants (6%) found the programs they watched uninteresting. In this case, the television system was confused with the functionality of the television channels, affecting the overall iTV experience:

But I’ll tell you, honestly... logically I don’t like TV.

Participant 15, RF group

#### Resistance Toward Video Calls

A few participants (8/32, 25%) did not like making video calls because they were either uncomfortable with seeing themselves on a screen (7/32, 22%) or perceived video calls as an intrusion into people’s privacy (1/32, 3%).

#### Financial Resources

Several participants (6/32, 19%) quickly raised the issue of the iTV’s price. Aside from the usefulness of the system, this aspect was quickly considered by the participants when they were planning to use the system:

What bothers me is the money, to change television.

Participant 14, NH group

Moreover, as soon as the question of a subscription was raised, the participants seemed reluctant.

#### Perceived Risks

The participants (10/32, 31%) showed a certain reluctance toward technology in general or toward the iTV in particular because risks, such as addiction and social barriers (6/32, 19%). Several participants felt that the use of technology represented a social barrier, intruding between people and making communication difficult:

But it’s frightening how many people have their smartphones and don’t look at the person next to them. It’s crazy.

Participant 1, home group

Also, 2 participants (6%) showed contempt or annoyance toward people who spend a lot of time on their phones. One participant feared becoming an addict and refused to learn how to use a digital tool:

There are dangers there, I have to be careful.

Participant 8, NH group

Finally, 1 participant feared that iTV would be used as an excuse to reduce the number of carers or reduce the number of family visits.

Another barrier was excessive solicitation (4/32, 12%); some participants showed a certain resistance to ICTs (eg, social networks and iTV) because of oversolicitation. According to 1 participant, receiving text messages every day was inconceivable. Indeed, this form of remote communication is relatively recent:

No, no, no, once in a while! If people send me messages every day, it’s going to explode.

Participant 1, RF group

Oversolicitation could then lead to annoyance or even refusal to use the technology:

I don’t want that but that’s the flaw, it’s the instantaneity of today’s world.

Participant 12, home group

However, 1 participant mentioned the possibility of an evolution in this resistance with the evolution in the importance of certain values with age, that is, favoring social contact over entertainment:

Human contact is even more important at that age than seeing a documentary on which she falls asleep, eh.

Participant 12, home group

Another barrier was a lack of control over data (4/32, 12%); 2 (6%) participants mentioned the dangers of the internet and the importance of data protection. One of them was very reluctant to go online because someone she knew had been scammed. Generally speaking, the lack of control over technology seemed to bother the participants:

And can we deactivate this action? That’s the problem.

Participant 12, home group

### Anxiety Toward iTV Use

#### Overview

The majority reported no apprehension about using (25/32, 78%) or even making mistakes on the iTV (24/32, 75%; Figure 7). The few participants (7/32, 22%) who reported some anxieties were those who lived in RFs or NHs.

#### Anxiety-Provoking Technology

Several participants (14/32, 44%) reported feeling anxious when using their device: fear of making irreparable mistakes and getting stuck, having lost their bearings:

I’m not going to look too hard when it’s set, because I’m always afraid of going wrong anyway.

Participant 14, home group

Some participants (4/32, 12%) felt this apprehension every time they used their technology, while others (10/32, 31%) were especially stressed during the learning phase:

Computers really put me off because I can’t get the hand of it...Oh yes, I screw up... I lose things... Pfff.

Participant 11, home group

This apprehension was not the same depending on the technology: some participants (3/32, 9%) felt more comfortable with their computer than with their smartphone, with which they were less used to interacting, while others (2/32, 6%) apprehended using the television system:

I hope there’s no remote control.

Participant 3, home group

The remote controls seemed to generate stress because of their diversity (ie, each brand of television has a different remote control, as a participant stated: “Look, I don’t really like remote controls, but because they change all the time” [Participant 5, home group]); a large number of buttons; or the presence of generic buttons (eg, colored buttons with no symbols on it and arrows). During the test, 1 participant did not feel confident because of the workload required by the iTV functioning (ie, linking the information on the screen with that on the remote control). These anxious OAs did not necessarily show resistance, but they saw the iTV as inevitably more complicated to use, making it difficult to apply their digital literacy:

I do it all the time. I was looking for something more complicated.

Participant 11, RF group

To cope with this technological anxiety, some participants (4/32, 12%) adopted coping strategies such as neglect (eg, a participant preferred having someone operate her TV for her) or avoidance (eg, ignoring certain buttons or functions on their television and remote control), stated as follows:

Oh no, no, I just do the channel and that’s it. No, I haven’t explored it, and I don’t even know what these are [arrow buttons].

Participant 7, NH group

#### Anxiety-Provoking Context

One participant mentioned the influence of the context in which the technology was used on the stress felt at the time of use:

For train tickets for example, I don’t know... there’s something about it, there’s the urgency and the stakes.

Participant 5, home group)

According to her, what was most stressful was the stakes behind the successful use of the technology, as well as the potential consequences if it failed (eg, automated teller machine and train tickets). In the case of iTV, the ringtone used to notify the user of a call could also be stressful, with the user rushing to find the button to pick up the call (2/32, 6%).

#### Progressive Familiarization and Search Strategy

Some participants (11/32, 34%) were not afraid of pressing the wrong button and dared to explore. This confidence may have come from their own experience of computers or from their confidence in their ability to solve problems: they were aware that it was always possible to go back or ask for help:

Well, sometimes the experience consists of going back.

Participant 2, home group

You can go wrong at first, but you can go back, you can fix it.

Participant 10, RF group

The participants’ level of apprehension could then influence their strategies for finding solutions. If they found themselves stuck, some (5/32, 16%) opted for testing unknown or unused buttons, while others preferred to test randomly, seeing the mistake as an opportunity to learn something else. It is interesting to note that this confidence was acquired gradually, as OAs learned how the technologies worked and overcame their initial reluctance:

Now it’s easy, because I’ve learned, but before, no, it was a real barrier.

Participant 5, home group

### FC Dimension

#### Overview

Most participants (24/32, 75%) considered having support from their families or care staff (those living in NH or RF; Figure 8). The few respondents who did not call on family and friends in case of problems were those who lived at home (4/16, 25%) and in RFs (2/9, 22%). However, when it came to their ability to learn how to use a new device, the participants were more divided: of 32 participants, 8 (25%) were undecided, 12 (38%) were fairly confident, and 12 (38%) were not. Interestingly, the participants who were least confident about using a new device were those living in RFs.

#### Feeling of Self-Efficacy

As shown on the right of Figure 8, participants’ feelings of self-efficacy varied considerably. Some participants (12/32, 37%) were confident in their ability to use and solve problems on the iTV, as 3 (9%) of them pointed out the role of expert that they assumed among their friends and family. Therefore, autonomy in finding solutions (eg, consulting YouTube [Google LLC] tutorials and online forums) could be an indicator of a feeling of personal efficacy. It is interesting to note that the feeling of anxiety depended on the technology and remained variable with time. For example, a participant felt confident using the iTV but doubted their ability to use a tablet. Even before they started the test, other participants (12/32, 37%) were skeptical about their ability to use the iTV independently, as a participant stated, “I’ll need help with this” (Participant 2, RF group), and seemed to consider its use as very difficult or impossible:

I was looking for something more complicated.

Participant 11, RF group

One participant even questioned their place in this test, stating “I’m wasting your time, aren’t I?” [Participant 7, NH group]. This lack of confidence in their abilities could have influenced their first experience with the iTV in several ways. As participants used the iTV, mistakes could be more prominent than successes, as a participant stated, “Well, that’s a good start!” [Participant 9, RF group], and they tended to blame themselves (eg, slowness, lack of thinking, and impatience and haste) for the slightest problem they encountered, never questioning the design or functioning of the technology:

Well, maybe it’s because I’m not paying enough attention.

Participant 6, home group

The tests revealed the difference between participants who were actively looking for a solution and those who gave up at the first sign of difficulty and preferred to rely on the researcher’s help. Finally, a bias identified among some participants (4/32, 12%) was the supposed comparison with other people, younger or of the same age, making the situation even more frustrating:

I’m sure someone younger would have understood straight away.

Participant 14, home group

#### Occasional or Regular Help From Peers

In most cases (20/32, 62%), peers provided occasional assistance, in particular, to help participants in the event of a problem, to support them as they familiarize themselves with a new technology or feature, or to explore other functionalities:

My son told me “there’s a yellow line there.” He says you have to press really hard.

Participant 14, NH group

Training people in a new technology or function sometimes meant reassuring them during the first moments of interaction. On a few occasions, participants’ relatives were able to act as advisers, pointing out the following additional features that could meet their needs:

- Audio message to address difficulty in writing SMS text messages
- Subtitle option to address difficulty in understanding a television program
- Pause function on television to help with urinary problems

Finally, other participants could also benefit from regular help with administrative formalities. In this case, the relatives provided more than just help as they were doing things directly for the participants. Help often came from younger family members and occasionally from friends or digital advisers via local councils. For participants living in institutions, the care staff were able to absorb some of the family’s support role, especially when the family was not nearby. This feeling of trust between the resident and the professionals could facilitate the adoption of a new tool.

#### Peer Attitudes

Some participants (4/32, 12%) complained about the annoyance and impatience of their relatives:

My nephew often reproaches me: “You’re not watching! It’s indicated!”

Participant 16, RF group

Moreover, peers may have lost interest when faced with the participant’s resistance or may not be available to help if problems arise:

My family... they’ll tell me “I don’t have the time!”

Participant 15, home group

However, as 1 participant pointed out, the trainer’s attitude can be passed on to the learner and, therefore, influence their attitude toward the technology.

#### Peer Dependence

Several participants (10/32, 31%) depended entirely on their relatives for certain tasks (eg, video calls and tax declarations), which could accentuate the gap between their current skills and modern technological requirements:

For example, I have taxes, but my son does them, so I don’t even know how to do them myself.

Participant 11, home group

Depending on several people can constitute a barrier and complicate the day-to-day use of technology. For example, 1 participant was very frustrated and angry at having “lost” her computer when she moved into an institution, and another reported his dependence on his wife for the tablet they share. This dependence could be induced by peers or desired by the participants themselves. In the first case, peers might think that they were helping by sparing them the difficulties associated with the technology. This could be frustrating for 1 participant, who felt she was capable of managing her own accounts:

Because he’s afraid I don’t know how to manage my account, so he checks!...No, because I don’t need him, I do it myself.

Participant 16, RF group

In the second case, some participants tended to wait to be contacted or for someone to do it for them: although they had taken part in the test and shown their ability to use remote control, some participants living in NH (2/7, 29%) preferred to delegate its use to care staff, mainly because of the anxiety-provoking technology:

When they come, if the TV is on or off, they put it on for me or turn it off. I mean, I know how to turn it off, but still... I don’t feel safe using it.

Participant 14, NH group

### UC Dimension

#### Overview

The participants from the 3 settings appeared to be divided into 2 groups in terms of the perceived social pressure and their curiosity about technology (Figure 9). In contrast to participants living in RFs who perceived a strong influence from their relatives (6/9, 78%), participants living at home and in NHs seemed more divided.

Finally, participants living at home seemed to be more curious about the idea of discovering a new technology (13/16, 81%) compared to participants living in RFs and NHs, who seemed more nuanced.

#### Influence of an Exceptional Situation

In response to exceptional situations (eg, a relative moving abroad and the COVID-19 pandemic), peers may have adopted different behaviors in an attempt to maintain cohesion within the group of friends or family. For example, during the COVID-19 pandemic, several participants (11/32, 34%) mentioned using video calls to stay in touch with their loved ones and to take part in activities (eg, a multimedia café organized by a laboratory). As a result of these exceptional situations, certain functionalities such as video calls or text messages may have become the norm in terms of communication within the family, within a group of friends, or even in the professional environment (eg, association). The iTV could then have more added value since the COVID-19 pandemic and confinements, with the more widespread use of video calls:

If she’d had that [iTV with video calls], it would have made our lives a lot easier because we could have seen her. And it would have been nice for her, you know.

Participant 5, home group

#### Social Pressure

Both private and professional peers could have a direct influence on the use of a feature or technology by encouraging the use of particular apps (eg, Google Maps [Google LLC] to find the way home and Skype [Microsoft Corp] for remote meetings; 3/32, 9%). Relatives could also offer participants a technology (eg, a tablet, smartphone, or a connected watch), first perceiving it as a gadget and then, after a period of familiarization, discovering its usefulness in everyday life:

No, no, it [connected watch] was offered to me because... I said it was a gadget, but when I saw the applications…

Participant 2, home group

Furthermore, some participants (4/32, 12%) felt forced to use several apps depending on the habits of the person they were talking to (eg, Messenger [Meta Platforms], WhatsApp [Meta Platforms], and Signal [Signal Technology Foundation]):

I receive messages from people who each have the good idea of having a different application.

Participant 2, home group

They also felt oversolicited by their peers: depending on the generation, it is not usual to receive text messages every day or several times a day. However, this influence did not necessarily enable participants to become more skilled at using the features. In the case of video calls, 1 participant (3%) simply received the calls without really understanding how they worked:

He calls my sister, the kids, and we all see each other on the mobile. But I’m not the one doing it, I don’t know how.

Participant 11, home group

Finally, 2 (6%) participants pointed out their own influence on their mode of communication:

It would be the other way round...It’s easier for me to read than to listen. So, I prefer to have messages...

Participant 3, home group

They pointed out the frequency of exchanges with their loved ones:

And then I solicit them too, to get news.

Participant 7, NH group

#### Consequences of Noncompliance

Whether or not participants are resistant to ICTs (eg, video calls messages), they sometimes represent the only way for them to keep in touch with their loved ones living abroad (5/32, 16%):

I have children who live in Japan, so I only see them that way.

Participant 8, home group

For them, resisting these innovations would mean isolation and rejection by their peers. One participant felt excluded from his family following his refusal to use several technologies, and another felt ashamed of being out of step with her RF neighbors:

I told them I’m hopeless, I’m old-fashioned! So, they laughed because it can seem completely absurd nowadays.

Participant 9, RF group

Finally, 1 participant saw no alternative but to submit to the group norms:

Well, it’s not that they [the peers] push me, it’s mandatory.

Participant 5, home group

#### Temporary Group Compliance and Reactance

The adoption of certain modes of communication (eg, video calls) may have been only temporary, which is the time needed to overcome the exceptional situation that had prompted relatives to adopt them in the first place (eg, the COVID-19 pandemic and relatives moving abroad). Thus, the social pressure present at a given moment is not necessarily effective with time, especially if the participants were initially resistant to change or video calls (7/32, 22%):

Well, I’m the one who doesn’t want to....We used to do it during the COVID with the young persons who couldn’t come and see us. Otherwise, I don’t like it, I don’t like being seen, I don’t think it’s... for me anymore.

Participant 11, RF group

Although 1 participant communicated a lot via text messages, she seemed annoyed by the regular solicitations from her peers, thus provoking psychological reactance:

[People who are important to me push me to use messages and video calls] Yes, that’s true, but I don’t reply [laughs].

Participant 7, NH group

#### Computer Experience

Most participants (18/32, 56%) saw their previous experience with technology (eg, smartphone, computer, or tablet) as facilitating, if not essential, for using iTV. This technology seemed to partially use knowledge already acquired through other technologies (eg, icons and navigation), making it easier for people who already had certain reflexes and rudimentary knowledge to learn:

Yes, there are lots of gestures now that are new, that have been learned and that correspond.

Participant 9, home group

Certain symbols that were once incomprehensible (eg, menu and contact) have come to mean something, thanks to the use of technology in everyday life, and therefore, familiarity with technical vocabulary and overall operation:

But finally, I think it’s an icon [contact] that has become universal now, for everyone who uses a smartphone.

Participant 9, home group

However, some participants (3/32, 9%) stressed that it was not necessary to have highly developed digital literacy to use iTV:

It’s quite accessible to people who don’t have any skills, well, who don’t have a... how should I say... a culture.

Participant 16, home group

Sometimes, certain prior knowledge could even hinder the use of iTV, as in the case of 1 participant who had learned to press hard on the remote control buttons, thus causing several actions on the iTV instead of 1 action.

Some participants (2/32, 6%) pointed out the late access their generation had to the internet. Indeed, several participants (3/32, 9%) had acquired more or less advanced computer skills or even automatic skills with the arrival of computers in the workplace. However, the training received and the use of digital technology (whether at work or not) often date back several decades. So, with the rapid development of technology, 1 participant said she felt out of date:

Let’s just say it’s changed so much since then, it’s evolved so much that I’m sure I’m... [out of date].

Participant 16, RF group

However, 2 (6%) participants thought that the digital literacy of OAs would evolve rapidly in the future:

But I think that use will increase because, in NH, you are dealing with an age group that is of my generation and I belong to a generation that has had very little access to the internet.

Participant 8, home group

#### Technological Routine

In many cases (11/32, 34%), using the computer, tablet, or television was part of a well-established routine:

I’ve had this habit for a very, very, very long time.

Participant 4, home group

This routinized use could then lead to various events, such as the creation of habits that are difficult to disrupt. In addition, this routine was all the more difficult to change, and it was hard to establish with peers. Other problems included the development of automatisms, making it harder to transfer knowledge to other technologies:

I’m so used to [typing] 3, that’s it. But that’s to find the right button [on the remote control].

Participant 6, NH group

Another problems was the neglect of buttons not used on a daily basis:

As I only ever use the same buttons, there may be things I have that I don’t even look at.

H-14

#### Interest in Technological Innovations

Participants (14/32, 44%) seemed curious to discover the iTV functionalities and found it fun to try to figure out how the iTV worked, perceiving mistakes as challenges to be overcome.

Some participants (3/32, 9%) were rather curious about new technologies in general (eg, robots and computers), being interested in them since the early days of computing. The time and effort invested at the time would have made it easier for them to learn how to use a new technology:

So I bought some books, I didn’t understand anything... And I read and reread the same thing over and over again, and then that was it, I was able to design programs. That may also explain why, perhaps more quickly than others, I quickly understand how to use the software.

Participant 2, home group

Two (6%) participants emphasized the importance of initial motivation in the learning process, noting that imagining the potential benefits of technology can stimulate its exploration:

It can be exciting, in the sense that it’s great, I’m going to have something better and everything.

Participant 4, home group

Although curiosity seems crucial when learning to use the iTV, it may not be enough due to the apprehension of making irreparable mistakes or the lack of PU:

But I don’t see what it’s for, what it can lead to. Yes, I see it as a game.

Participant 10, RF group

#### Sensory Disorders

With advancing age, sensory problems (9/32, 28%; eg, visual, auditory, and touch) could appear and develop with time, limiting the use of certain technologies, such as smartphones (eg, difficulty typing on a small keyboard) and television (eg, visual fatigue). Participants who wore glasses to improve near vision generally did not wear them to watch television (11/32, 34%). In the case of iTV, these participants then had difficulty recognizing certain symbols on the screen and the remote control:

Oh maybe with glasses I’d see better.

Participant 10, home group

In terms of hearing, 1 participant with hearing aids felt the sound of the iTV was not loud enough: on a daily basis, he used headphones plugged directly into his television. With regard to touch problems, some participants (5/32, 16%) found it difficult to press certain buttons correctly (eg, arrows and back buttons) because of their design (ie, size and shape of the button) or their location (ie, in the middle of 2 buttons). One participant was convinced that she pressed the right button when, in fact, she pressed the wrong one:

That’s what I’ve pressed now, isn’t it? [Researcher] You pressed just below it.

Participant 14, NH group

#### Cognitive Disorders

With age, certain cognitive disorders can affect memory, information processing speed, and mental flexibility. During the test, some participants (15/32, 47%) could not remember and frequently asked for the function of buttons they had already used (eg, camera button). Performing the action once seemed insufficient for the information to be retained, which underlines the importance of reminding people on the screen of the buttons to use. Two participants (6%) complained that they were slow in thinking, learning, or even adapting to a new way of functioning:

You have to adapt, and it takes a bit longer to adapt now...You’re not as fast and your brain doesn’t work as quickly as it did a few years ago.

Participant 11, home group

Another manifestation of cognitive impairments was the false recognition of certain actions and buttons. Some participants (5/32, 16%) tried to remember a button they had supposedly already used instead of scanning the remote control again to identify the right button. Because of the difficulties mentioned above, 1 participant living at home could not imagine the iTV being used by people with cognitive impairments. Two (6%) others living in NHs felt that they had been in decline for some time, partly due to various events (eg, retirement, the COVID-19 pandemic, and heatwaves) also affecting the residents around them. Thus, 1 participant no longer considered herself “adapted” [Participant 6, NH group] to new technologies such as iTV, while another complained that her life had “shrunk” [Participant 7, NH group], giving her the feeling she “no longer had time to do anything” [Participant 7, NH group]. Finally, participants conscious of their cognitive decline and the impact on their performance questioned and blamed themselves more often for the difficulties encountered during the test:

Yes, but maybe if our brains were a bit more developed, we wouldn’t do anything stupid either.

Participant 16, RF group

#### Fatigability

As people age, they may become more fatigued, impacting the time spent on certain activities. Because of the effort required to discover the iTV (eg, gymnastics between the screen and the remote control, searching for information, and the number of steps in the scenario), some participants (8/32, 25%) apparently reached cognitive saturation, with increasing difficulty in maintaining their attention, refocusing after a distraction, or even blocking on a task:

The trick is to pay attention, you can’t do random things. You have to try and think, which isn’t always easy at our age.

Participant 10, RF group

The fatigue accumulated throughout the test may have hampered the use of the iTV and led to frustration if the participant got stuck on a task already completed:

My brain doesn’t want to work anymore.

Participant 4, RF group

For example, 1 participant gradually lost sight of the test objective, constantly wanting to return to the TV channels instead of following the instructions. By making repeated mistakes, 2 (6%) participants admitted confusing good and bad learning.

#### Attitude Toward Aging

Among the participants living at home, some seemed to perceive the onset of cognitive problems as imminent and inevitable. Beyond the participants’ current state of health, the image they had of aging and their perception of the time they had left in good health, or even to live, could influence their commitment to iTV, as well as its acceptability. One participant seemed impatient, declaring that he had no more time to lose with technologies that were complex to learn or that didn’t meet his needs:

This thing is a tool, so it has to provide me with the services I need quickly and immediately. I’ve got no time to lose in my life, I’ve got 89 anyway [laughs].

Participant 9, home group

Others did not see the point of learning to use the iTV, believing that they didn’t have much time left to enjoy it:

I’m in my 91s this year. So maybe I won’t get much out of it.

Participant 14, NH group

## Discussion

### Principal Findings

The main aim of this research was to contribute to the enrichment of existing models of acceptance of innovations by OAs. This study focused on the acceptability of an iTV system by 32 people living in different settings (eg, home, NH, and RF). A deductive qualitative analysis based on e-TAM identified 33 concepts, each related to the following themes: IU, PU, PEOU, UR, anxiety about the iTV, FCs, and UCs.

### Advantages of the Methodology Used

This qualitative research complements a previous study, currently being submitted, aimed at assessing the perceived usability and ability of OAs to use the iTV. The data extracted from these tests were self-reported and subject to certain biases. For example, some authors have shown the influence of stereotypes on the perceived ability to innovate [48], while others have discovered that OAs who reported using smartphones were in fact only using the basic functions of the phone (ie, making calls and sending SMS text messages) [49]. Therefore, using a purely quantitative study does not allow for a comprehensive understanding of the mechanisms behind the acceptance of technological innovations.

The deductive qualitative analysis used in this study is based on the Qualitative Analysis Guide by Leuven [47]. One of the advantages of this analysis method is the combination of approaches used. The transcripts were first analyzed separately, using a case-oriented narrative approach. This approach ensured that the interaction between the ideas from each transcript and the specific characteristics of the participants was not overlooked, particularly with the help of conceptual diagrams. Then, by cross-referencing all these ideas, more global concepts were developed. It was only at this point in the analysis that all the data were interpreted, allowing themes to be developed by drawing on the richness of the data and doing justice to the complexity of the experiences of the participants [50].

### Technology Paradoxes

This qualitative analysis has highlighted the different behaviors of OAs when they are confronted with the use of technological innovation. Participants (20/32, 62%) were both very curious about the functioning of iTV and anxious about having to use a television remote control. Some (15/32, 47%) said that they were already beyond the stage of being apprehensive about using technology, with 1 participant mocking these anxious people:

You’d think we were going to bring out the atomic bomb! “No, no, no, I don't want to touch it! It’s going to be dangerous!”

Participant 4, home group

It is interesting to note that these behaviors seemed to be influenced by different factors, such as the level of digital literacy or place of residence. In our study, the participants who lived in institutions (16/32, 50%) were also the ones who were the least familiar with the use of smartphones or computers. These results are in line with the literature on the use of technology by OAs [51-53] and highlight the nuances of older users’ experience when confronted with technological innovations.

In addition to current models of technology acceptance (eg, TAM and UTAUT), the authors have proposed a framework of technology paradoxes [22,23]. This model states that users, when using a technology, are subject to a certain number of paradoxes that create strong emotions, such as anxiety and stress. Wilson-Nash and Tinson [23] identified 3 types of paradox: functional, social, and psychological.

Among the functional paradoxes, the paradox of chaos versus control was the origin of several situations reported by participants and classified in the category of resistance. In the case of the participant complaining about the addition of functions on her television, she experienced the transition from a situation of control (ie, watching television channels) to a chaotic situation (ie, YouTube starting at the same time as the television), resulting in frustration and the stress of losing one’s bearings.

In order to deal with the emotions generated by paradoxes, users may adopt various coping strategies before or after acquiring technology to avoid them or confront them. Wilson-Nash and Tinson [23] have identified 5 coping strategies used by OAs: neglect, partnership, control, adjustment, and acceptance. The partnership strategy seems to have been used by some participants in this study (3/32, 9%) via a mechanism of humanization of the technology. The participants tended to consider the indications displayed on the left of the television screen as instructions to follow or even tips given by the iTV. The participants spoke of the “interrelationship” and mutual assistance that existed between them as users interacting with the remote control and the television screen:

View the photos, I press OK? Because they tell me to.

Participant 11, home group

Therefore, the personification of iTV observed by the researcher could prove to be a strategy for confronting the technology, enabling participants to overcome their anxiety when using the iTV. It would be interesting to investigate the humanization of technology further and to test whether reinforcing this feeling (eg, the presence of a virtual agent on the iTV) could influence the perceived usability and acceptability of the iTV.

Technologies are often perceived as isolating people from others, as evidenced by the risks perceived by the participants in this study. However, almost all participants (24/32, 75%) also admitted to calling on certain members of their family or friends to resolve problems encountered with their technology. Thus, mastering the technology, which is another adaptation strategy, also includes a social dimension.

The acceptance strategy put forward by Wilson-Nash and Tinson [23] states that OAs, because of their life experience and time perspective, have a greater tolerance of technology faults. In this study, the difficulties encountered and the time required to understand how the iTV worked did not seem to have a major influence on its perceived usability. Therefore, error tolerance may be a differentiating factor between generations, as may apprehension when learning how to use an iTV.

### Sense of Self-Efficacy and Performance

The feeling of self-efficacy refers to the evaluation of one’s personal abilities, not one’s personal worth [39]. Belief in one’s abilities can influence the level of effort put into using and learning the technology, as well as thoughts and emotional reactions when using the technology [54]. Thus, the fact of not believing in one’s abilities, or even failing several times, does not normally influence one’s self-esteem but, on the contrary, can greatly harm motivation and performance. In this study, by blaming themselves for the difficulties they encountered, the participants tended to feel ashamed of not succeeding and even devalued themselves:

So turning it off, I don’t know where it is. That’s what I told you, eh, I’m ashamed but... [laughs].

Participant 9, RF group

In this case, the perceived inability to use an iTV seemed to influence self-esteem. Furthermore, Arning and Ziefle [55] stated that when a device presents technical difficulties, OAs experience a loss of technical confidence and begin to question their own value. These elements refer to the paradox of competence versus incompetence proposed by Wilson-Nash and Tinson [23], where users can feel both a sense of accomplishment when using technology successfully and a sense of self-doubt when encountering difficulties.

Authors have also found that OAs who consider themselves targeted by a stereotype tend to reduce their capacity for innovation (ie, their ability to mobilize their resources) to avoid appearing incompetent [48]. Therefore, agist stereotypes may indirectly influence older users’ feelings of efficacy.

Another factor that can influence OAs’ sense of self-efficacy is institutionalization, which is often associated with an increase in the degree of dependency of OAs. An institutionalization syndrome is characterized by apathy, indifference, reduced cognitive ability, difficulty in expressing feelings, and loss of autonomy [56]. The lives of residents in geriatric institutions are routinized to facilitate the organization of care, implying that residents may lose control over their activities. However, residents who are given control over their daily lives are more socially active, more involved in activities, happier, healthier, and live longer than those who are kept staff-dependent [57,58]. Nevertheless, beyond the possibility of exercising control, the desire for personal control tends to diminish with age, as does self-esteem and the belief in efficacy [39]. One participant in this study, living in an NH, relied heavily on the care staff to use her television. Although she took part in the test and showed that she was capable of using television, she preferred to delegate its use on a daily basis, in particular, because it made her anxious. Other levers exist, such as the presence of a role model (ie, a person with whom we can identify [age and sex] and who is able to manage) or resorting to social persuasion (ie, support and encouragement from carers and care staff) [39].

### The Influence of Motivation on the Acceptability of ICTs

Several participants (3/32, 9%) stressed the influence of motivation in the acceptance of a technology or feature. According to the literature, there is an essential distinction between voluntary behavior and behavior resulting from external pressure or control [59]. Intrinsically motivated behaviors are performed because of interest and to satisfy innate psychological needs for competence and autonomy, whereas extrinsically motivated behaviors are often carried out for external and instrumental reasons.

In the literature, enjoyment has often been used to represent this notion of intrinsic motivation [60]. Therefore, the enjoyment experienced when using a technology could reduce the perception of the effort made during its use [61] and thus be a predictor of the IU [60]. In this study, some participants (3/32, 9%) expressed curiosity about the iTV and emphasized the playful nature of its handling, taking a certain enjoyment in discovering its functioning. What was categorized as an interest in technological innovations could, therefore, represent a source of intrinsic motivation for the participants. This study appears to align with existing literature, although there are certain limitations to the concept of enjoyment. Indeed, while the use of the iTV could be enjoyable, some participants (13/32, 41%) saw no point in using it on a daily basis. Therefore, PU remains an essential factor in the long-term acceptance of technology.

Another aspect raised in this study is the extrinsic motivation to use a new technology. Some participants (4/32, 12%) mentioned a certain pressure to learn how to use a computer, for example, with the digitization of administrative services (eg, train e-tickets and tax). Indeed, 1 participant said that under normal circumstances, she would never have explored a new technology on her own. However, although this digitalization is pushing OAs to acquire digital knowledge, this learning process remains costly. The obligation, combined with the difficulty of increasing skills and knowledge, seemed to irritate these participants. Indeed, to avoid constantly disturbing her family and friends, 1 participant put a certain amount of pressure on herself to solve her problems on her own:

They seem to say that we all have to learn and manage. But it’s not... it’s not easy, is it?

Participant 11, home group

### Limitations

This study had several limitations, such as the small number of user tests conducted. The small sample in the study prevented us from generalizing the results to OAs living at home, in NH, and in RF.

Another limitation was the recruitment method for OAs living at home. Owing to technical and logistical constraints, user tests with these participants were conducted solely in the Paris region, while tests with OAs in NHs and RFs primarily took place in rural areas of the Grenoble region.

The final limitation of the study was the influence of experimenter bias, which could either negatively impact or inadvertently enhance participants’ acceptance of the iTV. Throughout the scenarios, the researcher was able to provide clues on how to use the iTV, if the participants asked for them, if they were blocked, or if they made a mistake. The presence of the researcher in this supportive role could then help the participants to overcome the problems encountered more easily, thereby reducing the perceived difficulty of use and anxiety:

[I felt confident when I used e-lioTV] Yes, but because you were there, sweetheart!... Otherwise, it would have gone out of the window.

Participant 4, RF group

However, the presence of the researcher could also increase anxiety, placing the participants in a situation of evaluation:

It's the setting in a way. It's an exercise, I have to pass, so there's stress.

Participant 9, home group

And I don't know... You're destabilizing me [laughs].

Participant 7, NH group

### Future Work

This research was inspired by the user-testing method, which is rooted in user-centered design. This method enabled us to observe the behavior of OAs, as they familiarized themselves with the iTV, and to identify the factors influencing its use. However, this evaluation was a time evaluation and did not allow us to monitor and understand changes in acceptance with time. A future study could focus on the long-term use of the iTV, specifically examining how the influence of the factors identified in this research evolves with time. It would be valuable to investigate whether these factors continue to affect acceptance of the iTV after several months of use. Moreover, the e-TAM developed in this study was based on different models from the literature (eg, TAM and Senior TAM). Another perspective could be to incorporate concepts from user experience research to propose a unified model.

### Recommendations

#### Overview

Conducting user tests with OAs living in different living environments requires techniques to be personalized and adapted, in particular, to compensate for their frailty and low levels of self-efficacy and digital literacy [62]. The following recommendations seek to share best practices for implementing user-centered design methods with OAs. They are intended for researchers and companies aiming to develop user-friendly technologies tailored to OAs.

#### Prioritize the Use of Codiscovery Learning

The think-aloud method requires users to express their thoughts, feelings, and actions while interacting with a tool. During the user tests, we observed that OAs struggled to verbalize their experiences while using the iTV, often forgetting to do so altogether. This is consistent with findings in the literature indicating that concurrent think-aloud practice induces greater cognitive load and stress in older participants, thus negatively affecting their performance [63-65]. Therefore, researchers should prioritize codiscovery learning, where users collaborate in teams. This approach encourages them to verbalize their thought processes while interacting with one another to complete tasks [64].

#### Account for Desirability Bias in Researcher-Participant Interactions

During user tests, participants often feel that they are being evaluated, leading them to adopt behaviors that present them in a positive light. This social desirability bias can result in excessively positive feedback about the device, even in the face of clear usability limitations. Among the various tactics adopted by participants, some may portray themselves as weak or dependent to elicit assistance, for example: “So, uh, I don’t know how to do this... As I said, I’m not good at this!” (Participant 9, RF group). In such cases, researchers can respond to each question with another question and reassure the participant that they are on the right track [62]. This technique, integrated into the protocol of this study, enabled some participants with lower self-efficacy to successfully complete each scenario.

### Conclusions

This study aimed to describe in detail the factors influencing iTV acceptance among OAs living in different settings (home, NH, and RF), using the example of e-lioTV. A total of 32 OAs used the communication functionalities of the iTV system (ie, messaging and video calling) and shared their opinions about the learning process, daily use, and iTV adoption. On the basis of the e-TAM built on the technological acceptance literature, 33 concepts were identified among the 7 determinants of iTV use (IU, PEOU, PU, UR, anxiety toward iTV use, FCs, and UCs). No new determinant was found during the qualitative analysis, even though some factors (eg, FCs and UCs) were completed and extended to consider all the nuances of OAs’ experience when interacting with an iTV system.

To conclude, the iTV acceptance seemed to be context, technology, and characteristic dependent. The participants seemed to agree with recommending the iTV to others; they also found using the iTV relatively easy and pleasant, and thus, they felt confident when using it. Almost all participants considered having support from their family and friends to learn or use a new technology. However, the participants had a divided opinion on iTV IU, the feeling of self-efficacy, and social pressure.

It is also interesting to point out the dimensions that generated the most disagreement between the institutionalized participants and those living at home. While participants living at home seemed to be quite consistent on some dimensions, the institutionalized participants shared more nuanced opinions about the usefulness of the iTV, the effort required to learn how to use it, the resistance to iTV, and the apprehension about pressing the wrong button. Finally, persons living at home were more curious about and interested in technological innovations than other participants.

## Abbreviations

e-TAM: extended technology acceptance model
FC: facilitating condition
ICT: information and communication technology
iTV: interactive television
IU: intention to use
NH: nursing home
OA: older adult
PEOU: perceived ease of use
PU: perceived usefulness
RF: residential facility
TAM: technology acceptance model
UC: user characteristic
UR: user resistance
UTAUT: unified theory of acceptability and use of technology
